# Amino Acid- vs. Peptide-Odorants: Responses of Individual Olfactory Receptor Neurons in an Aquatic Species

**DOI:** 10.1371/journal.pone.0053097

**Published:** 2012-12-27

**Authors:** Thomas Hassenklöver, Lars P. Pallesen, Detlev Schild, Ivan Manzini

**Affiliations:** 1 Department of Neurophysiology and Cellular Biophysics, University of Göttingen, Göttingen, Germany; 2 Cluster of Excellence “Nanoscale Microscopy and Molecular Physiology of the Brain” (CNMPB), University of Göttingen, Göttingen, Germany; Monell Chemical Senses Center, United States of America

## Abstract

Amino acids are widely used waterborne olfactory stimuli proposed to serve as cues in the search for food. In natural waters the main source of amino acids is the decomposition of proteins. But this process also produces a variety of small peptides as intermediate cleavage products. In the present study we tested whether amino acids actually are the natural and adequate stimuli for the olfactory receptors they bind to. Alternatively, these olfactory receptors could be peptide receptors which also bind amino acids though at lower affinity. Employing calcium imaging in acute slices of the main olfactory epithelium of the fully aquatic larvae of *Xenopus laevis* we show that amino acids, and not peptides, are more effective waterborne odorants.

## Introduction

Amino acid odorants are widely used olfactory stimuli for aquatic vertebrates like fish [1]–[4], amphibia [5]–[7], as well as aquatic invertebrates [8]–[10]. As protein decomposition, in particular food decomposition, generates amino acids, these stimuli have been proposed to serve as cues in the search for food [11]–[13]. Olfaction in vertebrates begins with the binding of odorants to olfactory receptors (ORs) located on cilia or microvilli of olfactory receptor neurons (ORNs) situated in the olfactory epithelium (OE). The activation of ORs triggers the activation of G-proteins, which in turn initiate transduction cascades generally leading to depolarization of the ORNs and to receptor potentials (for a review see [14]). The ORs for amino acid detection are as yet, with few exceptions [15], [16], unknown, and the concentrations of amino acids that have been used to stimulate individual ORNs were rather high in some physiological studies (e.g. [3], [5], [6], [8], [9], [17], [18]). Furthermore, it is known that protein decomposition also generates a considerable amount of soluble peptides [19]. Also, except for a study in the rainbow trout by Hara [20], and with the exception of peptide ligands of major histocompatibility complex (MHC) molecules [21], [22], to the best of our knowledge peptide odorants have so far not been tested in aquatic species. One might thus question whether amino acids are the natural and adequate stimuli for the ORs they bind to. Alternatively, these receptors could be peptide receptors which also bind amino acids though at lower affinity. There are a number of endogenous peptides with specific physiological roles. *N*-Acetylaspartylglutamic acid (NAAG) is, for instance, the most abundant dipeptide in the brain [23], activating a specific receptor, the metabotropic glutamate receptor type 3 [24], [25]. Other well known examples of endogenous peptides are, e.g. the thyrotropin-releasing hormone (TRH), and its receptor [26], or the opioid peptides and their receptors [27]. It is thus by no means excluded that ORs that are commonly called amino acid receptors do bind peptides at higher affinity and that their binding of amino acids is a non-specific side effect.

Here we analyse whether di- and tripeptides elicit comparable or stronger olfactory responses in amino acid-sensitive ORNs. The result is largely negative with one interesting exception, which allows to speculate about the binding properties of amino acid odorants at their specific OR.

## Materials and Methods

### Preparation of acute slices of the olfactory epithelium

Larval *Xenopus laevis* (stages 51 to 54; staged after [28] were chilled in iced water and then killed by transection of the brain at its transition to the spinal cord, as approved by the Göttingen University Committee for Ethics in Animal Experimentation. A block of tissue containing the OE, the olfactory nerves and the anterior part of the brain was dissected. The tissue was then glued onto the stage of a vibroslicer (VT 1200S, Leica, Bensheim, Germany), covered with bath solution (see below) and cut into 120–130 µm thick horizontal slices.

### Solutions, staining protocol and stimulus application

Standard bath solution consisted of (in mM): 98 NaCl, 2 KCl, 1 CaCl_2_, 2 MgCl_2_, 5 glucose, 5 Na-pyruvate, 10 HEPES, 230 mOsmol/l, pH 7.8. As control odorant stimulation, we used amino acids (L-arginine, glycine, L-lysine, L-methionine), which were either applied separately (each at a concentration of 200 µM) or as a mixture (L-arginine, L-lysine and L-methionine; each at 200 µM). All amino acids and bath solution chemicals were purchased from Sigma (Deisenhofen, Germany). Peptides consisting of selected combinations of L-arginine, L-methionine, L-lysine (group I peptides) and L-arginine, L-methionine, glycine (group II peptides) were purchased from GenScript (Piscataway, NJ, USA; L-arginyl-L-methionine, L-methionyl-L-arginine, L-arginyl-L-methionyl-L-arginine, L-methionyl-L-arginyl-L-methionine, L-arginyl-L-lysine, L-lysyl-L-arginine, L-arginyl-L-lysyl-L-arginine, L-lysyl-L-arginyl-L-lysine, glycyl-L-arginine, L-arginyl-glycine) or Sigma (L-methionyl-glycine, glycyl-glycine, glycyl-glycyl-glycine). Tissue slices (see above) were transferred to a recording chamber, and 200 µl of bath solution containing 50 µM Fluo-4/AM (Molecular Probes, Leiden, The Netherlands) was added. Fluo-4/AM was dissolved in DMSO (Sigma) and Pluronic F-127 (Molecular Probes). The final concentrations of DMSO and Pluronic F-127 did not exceed 0.5% and 0.1%, respectively. Cells of the OE of larval *Xenopus laevis* express multidrug resistance transporters with a wide substrate spectrum, including Ca^2+^-indicator dyes [29], [30]. To avoid transporter-mediated destaining of the slices, 50 µM MK571 (Alexis Biochemicals, Grünberg, Germany), an inhibitor of multidrug transporters, was added to the incubation solution. The preparations were incubated on a shaker at room temperature for 35 minutes. During the experiment, the recording chamber was constantly perfused with bath solution applied by gravity feed from a storage syringe through a funnel drug applicator. The flow rate was 350 µl min^−1^. The tip of the applicator was placed directly above the OE. Before starting the experiments the slices were rinsed with bath solution for at least five minutes. After the first ten frames of each recording, amino acids and peptides were applied into the funnel in random order without stopping the bath solution flow. Bath solution was removed from the recording chamber through a syringe needle placed close to the OE. All experiments were conducted at room temperature. The reproducibility of peptide responses was verified by regularly repeating the application at least twice. To ensure sustained cell viability amino acids as positive control were regularly applied during and at the end of all experiments. The minimum interstimulus interval was at least two minutes in all of the experiments.

### Ca^2+^ imaging and data evaluation

Changes of intracellular calcium concentrations of individual ORNs were monitored using a laser-scanning confocal microscope (LSM 510/Axiovert 100 M, Zeiss, Jena, Germany). Fluorescence images (excitation at 488 nm; emission >505 nm) of the OE slice were acquired at 1.27 Hz and 786 ms exposure time per image. The thickness of the optical slices excluded fluorescence detection from more than one cell layer. The data were analyzed using custom written programs in MATLAB (Mathworks, Natick, USA). To facilitate selection of regions of interest, a ‘pixel correlation map’ was obtained by calculating the cross-correlation between the fluorescence signals of a pixel to that of its immediate neighbors and then displaying the resulting value as a grayscale map. As physiological responses often give similar signals in adjacent pixels, this method specifically highlights those pixels. In contrast, pixels that contain only noise show uncorrelated traces and thus appear dark in the cross-correlation map [31]. The fluorescence changes for individual regions of interest, i.e. individual ORNs, are given as ΔF/F values. The fluorescence changes ΔF/F were calculated as ΔF/F = (F – F_0_)/F_0_, where F was the fluorescence averaged over the pixels of an ORN, while F_0_ was the average fluorescence of that ORN prior to stimulus application, averaged over three images [32]. A response was assumed if the following criteria were met: (i) the maximum amplitude of the calcium transient had to be higher than the maximum of the prestimulus intensities; (ii) the onset of the response had to be within ten frames after stimulus application. Statistical significance was determined by either paired or unpaired t-tests (see also respective Figure legends).

## Results

**Figure 1:**
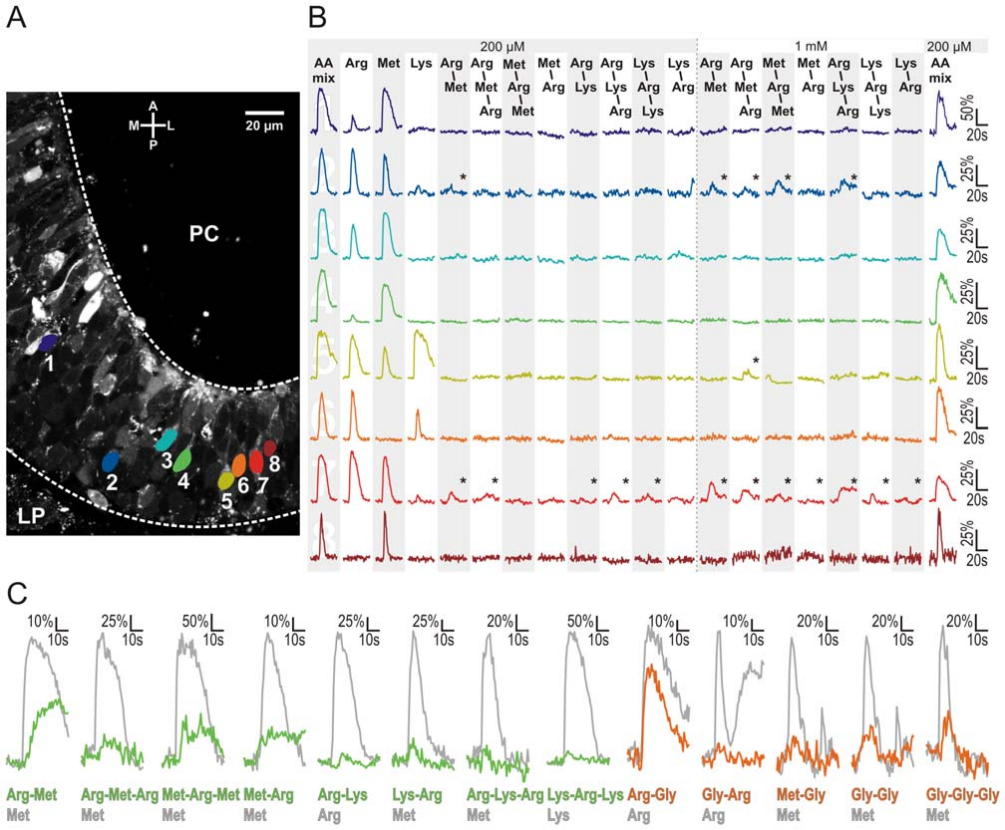
Amino acid- and peptide-induced changes in calcium-dependent fluorescence of individual ORNs in slices of the olfactory epithelium. (A) Slice preparation of the OE of larval *Xenopus laevis* stained with Fluo-4 AM. The colored ovals (#1–#8) indicate the eight ORNs that were responsive to the mixture of amino acids. (B) Time courses of [Ca^2+^]_i_ transients of the eight ORNs marked in A, elicited by application of amino acids (L-arginine, L-methionine and L-lysine as a mixture or singularly; each at a concentration of 200 µM) and peptides (consisting of L-arginine, L-methionine and L-lysine; 200 µM and 1 mM). Discernible peptide induced [Ca^2+^]_i_ transients are marked by an asterisk. To check for ORN viability, the mixture of amino acids was applied at the end of the experiment. (C) Examples of peptide induced calcium transients originating from different ORNs (group I peptides, green, L-arginyl-L-methionine (Arg-Met), 5 mM; L-arginyl-L-methionyl-L-arginine (Arg-Met-Arg), 1 mM; L-methionyl-L-arginyl-L-methionine (Met-Arg-Met), 1 mM; L-methionyl-L-arginine (Met-Arg), 5 mM; L-arginyl-L-lysine (Arg-Lys), 200 µM; L-lysyl-L-arginine (Lys-Arg), 1 mM; L-arginyl-L-lysyl-L-arginine (Arg-Lys-Arg), 1 mM; L-lysyl-L-arginyl-L-lysine (Lys-Arg-Lys), 1 mM;; group II peptides (see Material and Methods), orange, all applied at 200 µM). As reference also the highest amino acid-induced (200 µM) calcium transient is depicted. [AA mix: amino acid mixture].

**Figure 2:**
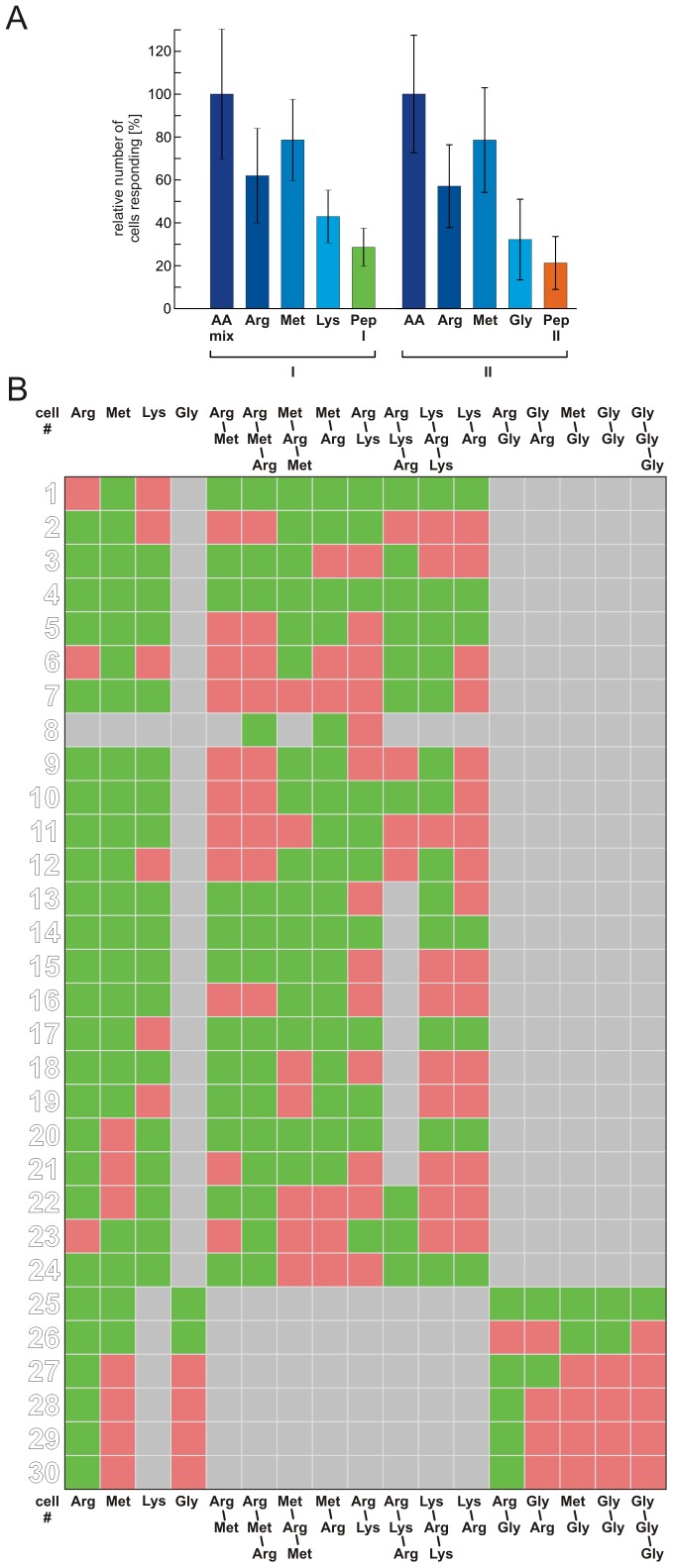
Response profiles of ORNs to amino acid and peptide stimulation. (A) Relative number of amino acid-sensitive ORNs reacting to individual amino acids (200 µM) or at least to one of the thirteen tested peptides. Only a fraction of amino acid-responsive ORNs also responded to group I peptides (1 mM, 12 of 42 ORNs in four slices) or group II peptides (200 µM, 6 of 28 ORNs in four slices). The fraction of ORNs sensitive to group I peptides did not differ from the fraction of ORNs sensitive to group II peptides. (B) Response matrix of all peptide-sensitive ORNs to the applied stimuli (green, response to applied stimulus; red, no response; grey, not tested; applied peptide concentration: ORN #1–#12, 1 mM; ORN #13–#21, 5 mM; ORN #22–#24, 10 mM; ORN #25–#31, 200 µM). [AA mix: amino acid mixture, AA: amino acids, Arg: L-arginine, Met: L-methionine, Lys: L-lysine, Gly: glycine, Pep I: group I peptides, Pep II: group II peptides].

**Figure 3:**
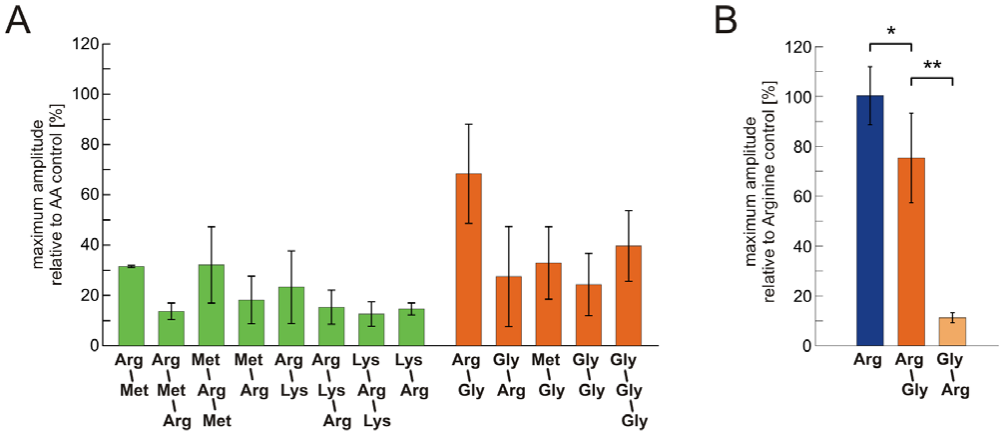
Peptide stimulation evokes calcium transients with lower maximum amplitude than stimulation with amino acids. (A) The maximum amplitude of [Ca^2+^]_i_ increases upon peptide application (green, group I, 1 mM; orange, group II, 200 µM) is much lower than upon application of amino acids (200 µM; number of responses averaged: L-arginyl-L-methionine (Arg-Met), 2; L-arginyl-L-methionyl-L-arginine (Arg-Met-Arg), 4; L-methionyl-L-arginyl-L-methionine (Met-Arg-Met), 9; L-methionyl-L-arginine (Met-Arg), 9; L-arginyl-L-lysine (Arg-Lys), 4; L-arginyl-L-lysyl-L-arginine (Arg-Lys-Arg), 7; L-lysyl-L-arginyl-L-lysine (Lys-Arg-Lys), 7; L-lysyl-L-arginine (Lys-Arg), 2; out of 12 ORNs, four OE slices; L-arginyl-glycine (Arg-Gly), 10; glycyl-L-arginine (Gly-Arg), 4; L-methionyl-glycine (Met-Gly), 4; glycyl-glycine (Gly-Gly), 4; glycyl-glycyl-glycine (Gly-Gly-Gly), 2; out of six ORNs, four OE slices). (B) Of the five group II peptides only the dipeptide L-arginyl-glycine (Arg-Gly) featured a stimulus-induced maximum amplitude of [Ca^2+^]_i_ increases comparable to stimulation with L-arginine (only ORNs exclusively sensitive to the amino acid L-arginine, i.e. #27–#30 taken into account). In contrast, the dipeptide glycyl-L-arginine (Gly-Arg) showed a weak response (averaging of multiple applications of glycyl-L-arginine (Gly-Arg); *, p<0.05; **, p<0.001, paired t-test, error bars represent standard deviation). [AA: amino acids, Arg: L-arginine].

**Figure 4:**
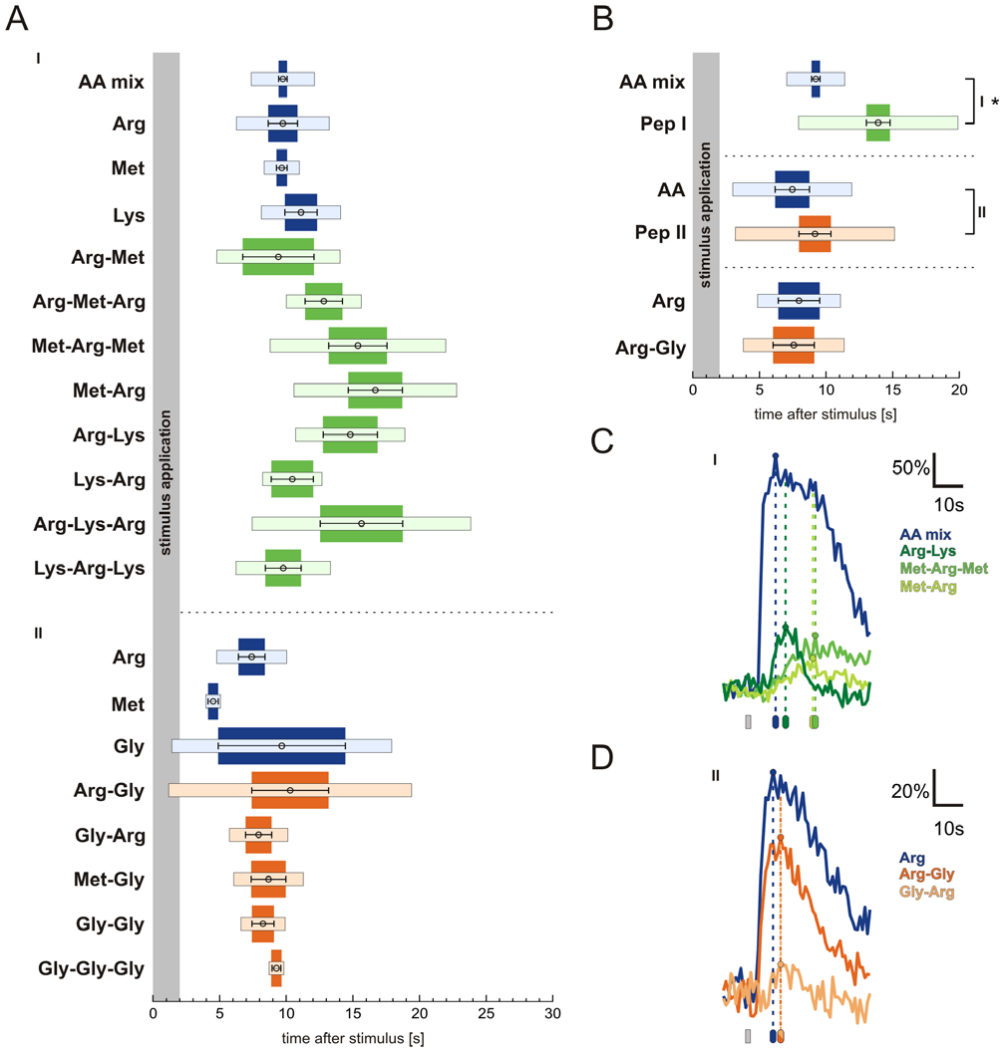
Group I and group II peptides elicit significantly different [Ca^2+^]_i_ transients in individual olfactory receptor neurons. (A) The mean time points of amino acid- and peptide-evoked calcium transient maxima varied for individual stimuli. Transients evoked by group I peptides show a tendency to reach their maximum amplitude later if compared to amino acid stimulations (green, group I peptides, 1 mM; number of responses averaged: AA mix, 67; L-arginine (Arg), 10; L-methionine (Met), 11; L-lysine (Lys), 6; L-arginyl-L-methionine (Arg-Met), 3; L-arginyl-L-methionyl-L-arginine (Arg-Met-Arg), 4; L-methionyl-L-arginyl-L-methionine (Met-Arg-Met), 9; L-methionyl-L-arginine (Met-Arg), 9; L-arginyl-L-lysine (Arg-Lys), 4; L-arginyl-L-lysyl-L-arginine (Arg-Lys-Arg), 7; L-lysyl-L-arginyl-L-lysine (Lys-Arg-Lys), 7; L-lysyl-L-arginine (Lys-Arg), 2; out of 12 ORNs, four OE slices; orange, group II peptides, 200 µM; number of responses averaged: L-arginine (Arg), 7; L-methionine (Met), 3; glycine (Gly), 3; L-arginyl-glycine (Arg-Gly), 10; glycyl-L-arginine (Gly-Arg), 4; L-methionyl-glycine (Met-Gly), 4; glycyl-glycine (Gly-Gly), 4; glycyl-glycyl-glycine (Gly-Gly-Gly), 2; out of six ORNs, four OE slices). (B) A combined analysis reveals that calcium transients evoked by applications of group I peptides show a significant delay of their maximum amplitude if compared to responses to the mixture of amino acids. In contrast, response maxima evoked by group II petides are not significantly shifted in comparison to amino acid controls. Even more clearly, response maxima evoked by L-arginyl-glycine (Arg-Gly) are not shifted if compared to maxima evoked by L-arginine in L-arginine-specific ORNs (not responsive to the other two amino acids L-methionine and glycine). Bars indicate standard deviation and error bars represent the standard error of the mean (*, p<0.0001; unpaired t-test, number of evaluated responses for the first group, AA mix: 67 responses, Pep I: 45 responses, 12 cells, four OE slices; for the second group, AA: 12 responses, Pep II: 25 responses, six cells, four OE slices; and for exclusively L-Arginine positive ORNs, Arg: four responses, Arg-Gly: six responses). (C) Typical responses upon application of amino acids and group I peptides. The maximum amplitude of [Ca^2+^]_i_ transients induced by group I peptides is smaller and shows a significant delay in comparison to [Ca^2+^]_i_ transients induced by amino acids. Circles and dotted lines indicate the maximum amplitude of each response. AA mix (200 µM, blue), L-arginyl-L-lysine (Arg-Lys; 1 mM, dark green), L-methionyl-L-arginyl-L-methionine (Met-Arg-Met; 1 mM, green), L-methionyl-L-arginine (Met-Arg; 1 mM, light-green). The odorant application is marked by a grey bar. (D) Representative example of [Ca^2+^]_i_ transients of an ORN sensitive to L-arginine (200 µM, blue), L-arginyl-glycine (Arg-Gly; 200 µM, orange) and glycyl-L-arginine (Gly-Arg; 200 µM, light-orange). Calcium signals evoked by L-arginyl-glycine showed the highest mean maximum amplitude of all tested peptides. In both peptide responses, the maximum amplitude is not shifted in comparison to the arginine application. [AA mix: amino acid mixture, AA: amino acids, Arg: L-arginine, Met: L-methionine, Lys: L-lysine, Gly: glycine, Pep I: group I peptides, Pep II: group II peptides].

We have analysed ORN responses to amino acid odorants and to peptide odorants consisting of these amino acids. We chose L-arginine, L-lysine, L-methionine and glycine, and a group of thirteen di- and tripeptides consisting of these amino acids (group I and group II peptides, see Material and Methods). Application of amino acids to acute slices of the OE, either as a mixture (each at a concentration of 200 µM) or individually (200 µM), induced transient increases of Ca^2+^-dependent fluorescence in several individual ORNs (Figure 1A). In the shown slice eight ORNs were responsive to amino acids. The exact response profiles to amino acids of these eight ORNs are shown in Figure 1B. Subsequent application of group I peptides, consisting of L-arginine, L-lysine and L-methionine, at an equal concentration of 200 µM elicited very faint responses in some of the amino acid-sensitive ORNs (Figure 1B). We did not notice peptide-induced responses in ORNs that were not responsive to amino acids in this nor in any other slice tested (data not shown). Subsequent application of group I peptides at a fivefold higher concentration (1 mM) only slightly increased the response amplitudes of ORNs that already responded at lower concentration. Furthermore, in some cases peptides that did not elicit responses at lower concentrations induced small responses if applied at a higher concentration (see Figure 1B). A further increase of the peptide concentration to 5 mM or 10 mM did not apparently increase the number of responding ORNs nor the amplitude of the responses (data not shown). Figure 1C shows ORN responses to amino acids and all thirteen peptides (group I peptides, green; group II peptides, consisting of L-arginine, L-methionine and glycine, orange). In total, we analysed responses of 70 ORNs (ten OE slices, ten animals; see Figure 2A). The data of these 70 ORNs were collected in two sets of experiments. In a first set of experiments we applied L-arginine, L-lysine and L-methionine and group I peptides. Of the 42 amino acid-responsive ORNs, 62% responded to L-arginine, 79% to L-methionine and 43% to L-lysine. As some ORNs responded to more than one amino acid, the frequencies sum up to values higher than 100%. A clearly smaller fraction of ORNs (29%) also responded to at least one of the eight group I peptides (Figure 2A). In a second set of experiments we applied L-arginine, L-methionine and glycine and group II peptides. In order to reduce the size and possibly the steric hindrance of the peptides at the receptor binding site, we chose to include glycine, the smallest amino acid found in proteins. In this second set, of 28 amino acid-responsive ORNs, 57% responded to L-arginine, 79% to L-methionine and 32% to glycine. As in the first set of experiments only a small subset of ORNs (21%) also responded to at least one of the five group II peptides (Figure 2A). The matrix in Figure 2B depicts the exact response profile of all peptide-sensitive ORNs. Figure 3A depicts the mean maximum amplitudes of the peptide-induced increases of Ca^2+^-dependent fluorescence relative to the amplitude reached upon application of amino acid controls. Out of the group I peptides (green bars), even the peptide that elicited the highest mean amplitudes (L-methionyl-L-arginyl-L-methionine) reached only about 32% of the amino acid-induced amplitudes. In comparison, the smaller group II peptides (orange bars), tendentially featured a slightly higher mean maximum amplitude. Thereby, the peptide L-arginyl-glycine showed an exceptionally high mean maximum amplitude. L-arginyl-glycine elicited responses in five of the six ORNs sensitive to group II peptides. Three of them were sensitive only to the amino acid L-arginine and the dipeptide L-arginyl-glycine (ORNs #28-30, see matrix in Figure 2B). Olfactory receptor neuron #27 showed an additional weak sensitivity to glycyl-L-arginine, and ORN #25 was sensitive to all applied stimuli. In Figure 3B we give a closer look at the four ORNs showing a specific amino acid sensitivity to L-arginine. Interestingly, in these ORNs the mean maximum amplitude of responses to the dipeptide L-arginyl-glycine was much higher than that of all other peptide responses (group II as well as group I), but with 75±6% still significantly lower than responses to the amino acid L-arginine alone. The reverse-substituted glycyl-L-arginine, however, showed only minor activity and the mean relative maximum amplitude was only 11±1%. An analysis of the time course of the calcium transients triggered by amino acids, group I and group II peptides gave heterogeneous results. Figure 4A shows the time points of the mean maximum amplitude of the responses to each of the applied odorants. Calcium transients evoked by group I peptides generally had a delay of their mean maximum amplitude if compared to those of amino acids. The mean time point of the maximum amplitude of all group I peptide responses showed a significant shift from 9.1±0.3 s (amino acids) to 13.7±0.9 s (peptides of group I) after stimulation (Figure 4B and C). In contrast, the time points of the mean maximum amplitude of the responses of all group II peptides did not significantly differ from those of amino acids [7.3±1.3 s (amino acids) vs. 9.0±1.2 s (peptides of group II); Figure 4B and D]. Interestingly, in the four ORNs specifically sensitive to L-arginine (see also Figure 3), the delay of the mean maximum amplitude for the L-arginyl-glycine (7.5±1.5 s; Figure 4B) was almost identical to that of L-arginine (7.9±1.5 s; Figure 4B).

## Discussion

It has long been known that fish as well as other aquatic vertebrates and invertebrates are able to smell amino acid odorants. This has been assessed in many studies that used a wide range of different neurophysiological techniques (extracellular recordings: [4], [33], [34], patch clamp: [3], [8], [9], [35], [36], calcium imaging: [2], [5], [6], voltage sensitive dyes: [37]). Behavioural studies have shown that amino acids are appetitive olfactory cues that elicit an attractive response [38]–[40]. The main sources of amino acids in sea and freshwater are: (i) direct release and excretion by the biota, (ii) bacterial exoenzyme activity, (iii) living cell lysis, (iv) decomposition of dead and dying autotrophic and heterotrophic organisms, and (v) release from biofilms [41], [42]. In natural aquatic environments the concentrations of dissolved free amino acids are normally very low. The reported concentrations generally range from <1 nM to about 70 nM in seawater [41], and from <1 nM to about 1 µM in estuarine waters and certain freshwaters [43]–[45]. Dissolved combined amino acids, most of them bound in small peptides, appear to typically occur in up to 10 times higher concentrations. They mainly consist of peptides and small proteins with molecular weights <1000 daltons [43], and are present in concentrations up to about 4 µM in seawater and up to 10 µM in natural freshwaters [41], [46]–[48]. As peptides with more than two amino acids are hydrolysed much faster than dipeptides [19], dipeptides are probably very abundant. This shows that in natural waters the concentration of dissolved free amino acids is generally much lower that the concentrations used to stimulate individual ORNs in neurophysiological experiments [3], [5], [6], [8], [9], [17], [18].

Based on this information and using the fully aquatic larvae of *Xenopus laevis* as a model system, we investigated the possibility that small peptides rather than amino acids are the natural olfactory stimuli for the ORs they bind to. In the first set of experiments we used L-arginine, L-lysine and L-methionine, as well as eight di- and tripeptides (group I peptides) consisting of these amino acids. These amino acids have previously been shown to elicit responses in ORNs of larval *Xenopus laevis* [6]. We found that di- and tripeptides are able to stimulate ORNs sensitive to amino acids the peptides consist of, although with a number of particularities: (i) only about one third of the ORNs that responded to amino acids responded to at least one of the eight peptides, (ii) the amplitudes of the peptide-induced [Ca^2+^]_i_ transients were without exception smaller than those induced by amino acids, and (iii) the peptide-induced calcium transients showed a delayed onset, a more gradual increase and significantly temporally shifted mean maximum amplitudes. Application of an up to 50 times higher peptide concentration did not significantly overcome these differences. These results led us to conclude that peptides are substantially less potent olfactory stimuli than amino acids. Importantly, we never observed peptide-induced responses in ORNs that were insensitive to amino acids. This excludes the presence of a further subpopulation of ORNs expressing ORs specific for peptide odorants. The ligand specificity of individual ORs has been shown to feature a high specificity for functional groups and molecular features, but in some aspects it also has a high degree of tolerance (for reviews see [49], [50]), i.e. individual ORs typically recognize a wide variety of structurally similar odorants. Taking into account these general features of ORs it is astonishing that the addition of only one or two amino acids to a free amino acid significantly disrupts its binding to the OR. A number of ORNs of larval *Xenopus laevis* have been shown to be broadly tuned to amino acid odorants [6], which might suggest that some ORs recognize the main functional groups of amino acids. However, as these functional groups are present also in di- and tripeptides, some other factor must account for the significantly lower responses to peptides. Steric hindrances due to the larger size of peptides could disrupt the binding of the odorant to the OR. For mammalian ORs it has been shown that not only functional groups, but also the carbon chain length of aliphatic odorants changes their binding characteristics to ORs [51], [52]. The second set of experiments aimed at verifying this hypothesis. We employed L-arginine, L-methionine and glycine and a group of five di- and tripeptides (group II peptides) consisting of these amino acids. We substituted L-lysine with glycine, which has just a hydrogen atom as side chain, to minimize the size of the resulting di- and tripeptides. As expected from a previous study [6], a lower number of ORNs responded to glycine if compared to the other individual L-amino acids used in the present study. One fifth of the ORNs that responded to amino acids also responded to at least one of the group II peptides. As the group I peptides, also the group II peptides did not induce responses in ORNs that were insensitive to amino acids. In contrast, the response time courses induced by group II peptides, in respect to the delay of the mean maximum amplitude, were not different from those induced by amino acids. But responses to nearly all group II peptides showed similarly low amplitudes as responses to group I peptides. Only L-arginyl-glycine elicited a significantly higher response than all other peptides used in this study.

The main outcome of this study is that free amino acids and not peptides, although more abundant in natural aquatic environments [41], [43]–[45], are generally more effective odorants (but see responses to L-arginyl-glycine). A similar conclusion was drawn in a study in the rainbow trout [20]. In contrast to our present study, Hara recorded summed extracellular recordings from a defined part of the dorsal olfactory bulb upon mucosal odorant application. Therefore, this study could neither exclude peptide responses being mapped in other parts of the olfactory bulb, nor could it draw conclusions on response characteristics of individual ORNs. Threshold concentrations for different free amino acids obtained recording summed stimulus-evoked activity on many neurons (electro-olfactogram, electro-encephalogram or olfactory nerve recordings), have been reported to range between 1 nM and about 10 µM [34], [53], and dose response relationships for amino acid odorants have been reported to have broad dynamic ranges, covering 6–7 log units [54]. This is rather surprising, given the very low concentrations of free amino acids in natural waters, generally in the low nanomolar range [41], [43]–[45]. Concentrations of amino acids generally used to stimulate individual ORNs (patch clamp and calcium imaging) are usually much higher [3], [5], [6], [8], [9], [17], [18]. The threshold concentrations for free amino acids of individual *Xenopus* ORNs determined in a calcium imaging study have been reported to range from 200 nM to 200 µM [55]. In behavioural experiments the employed free amino acid concentrations are in the same range [38]–[40]. This suggests that some recording techniques might not be sensitive enough to detect the effective threshold concentrations of odorant molecules. On the other hand it is known that the convergence of many ORN axons onto a single glomerulus in the olfactory bulb shifts the odorant thresholds towards lower concentrations [56], [57]. This amplification step suggests that the sensitivity of the olfactory system is higher as the sensitivity of its individual ORNs.

The results of the present study also allow to speculate about binding properties of amino acid odorants at their specific ORs. In this context, the ORNs that showed specific amino acid sensitivity to L-arginine are of particular interest. These ORNs were strongly sensitive also to the dipeptide L-arginyl-glycine, but neither showed a comparable strong response to glycyl-L-arginine nor to the other peptides or amino acids. This suggests that the successful activation of the OR expressed by these ORNs requires intact and properly positioned α-carboxyl and α-amino groups and that also the amino acid side chain plays an important role. The dipeptide L-arginyl-glycine featuring the L-arginine-specific side chain, but, due to the peptide bond between L-arginine and glycine, having a slighly displaced α-carboxyl and α-amino groups, still strongly activates the OR. In contrast, glycyl-L-arginine, with reversed α-carboxyl and α-amino groups, did not or only faintly activate this OR (see Figure 3B and Figure 4D). In fish, relatively independent receptor sites for basic amino acids, particularly for L-arginine, have already been suggested a few decades ago by a number of cross-adaptation studies [58], [59] (see also [53]). More recently, a goldfish OR tuned to basic amino acids has been characterized in a study by Speca and coworkers [15]. In *Xenopus,* an olfactory receptor preferentially responding to basic amino acids has been described by Mezler and coworker [16], while ORNs with exclusive sensitivity to L-arginine have been reported in a previous study of our group [6]. The latter study revealed about 5% of all amino acid-sensitive ORNs to be exclusively sensitive to L-arginine.

Together, the data presented here clearly show that amino acids rather than small peptides are the adequate stimuli of a subgroup of ORs of larval *Xenopus laevis.* Future studies will be necessary to validate this conclusion for other aquatic species. The present study also suggests that the amino acid-specific ORs of *Xenopus* might be well-suited to investigate binding properties of odorants at ORs with identified response profiles.
